# Contributions of Robotics to the Safety and Efficacy of Invasive Monitoring With Stereoelectroencephalography

**DOI:** 10.3389/fneur.2020.570010

**Published:** 2020-12-16

**Authors:** Amir H. Faraji, Madison Remick, Taylor J. Abel

**Affiliations:** ^1^Department of Neurological Surgery, Houston Methodist Hospital, Houston, TX, United States; ^2^Department of Neurological Surgery, University of Pittsburgh, Pittsburgh, PA, United States; ^3^Department of Bioengineering, University of Pittsburgh, Pittsburgh, PA, United States

**Keywords:** robotics, stereoelectroencephalography, frameless technique, epilepsy surgery, neurosurgery

## Abstract

The purpose of this review is to provide a discussion of the history and utility of robotics in invasive monitoring for epilepsy surgery using stereoelectroencephalography (sEEG). The authors conducted a literature review of available sources to describe how the advent of surgical robotics has improved the efficacy and ease of performing sEEG surgery. The sEEG method integrates anatomic, electrographic, and clinical information to test hypotheses regarding the localization of the epileptogenic zone (EZ) and has been used in Europe since the 1950s. One of the primary benefits of robot-assisted sEEG implantation techniques is the ability to seamlessly transition between both orthogonal and oblique trajectory types using a single technique. Based on available information, it is our view that, when applied appropriately, robotic sEEG can have a low rate of complications and many advantages over both non-robotic sEEG implantation and traditional craniotomy-based invasive monitoring methods.

## Introduction

The utilization of surgical robots has improved the precision and accuracy of a given procedure. Robots have predetermined, reproducible, and exact paths that limit error, excursions, and the potential for injury to nearby structures if utilized properly (1). Neurosurgeons recognized the utility of robotic assistance more than 30 years ago with the Minerva CT-guided biopsy (University of Lausanne) and PUMA (Advance Research and Robotics) systems, introduced in 1985 (2–4). These systems demonstrated high rates of malfunction and safety concerns related to a lack of operational safeguards and clinical experience. Additional operative robotic systems were subsequently introduced, including the NeuroMate (Integrated Surgical Systems) in 1987, an MRI-compatible system in 1995, and the Cyberknife system (Accuray Incorporated) in 1998 (5–7). The utilization of surgical robotics has improved the utility and performance of several neurosurgical procedures (3, 5, 8–16).

**Figure 1:**
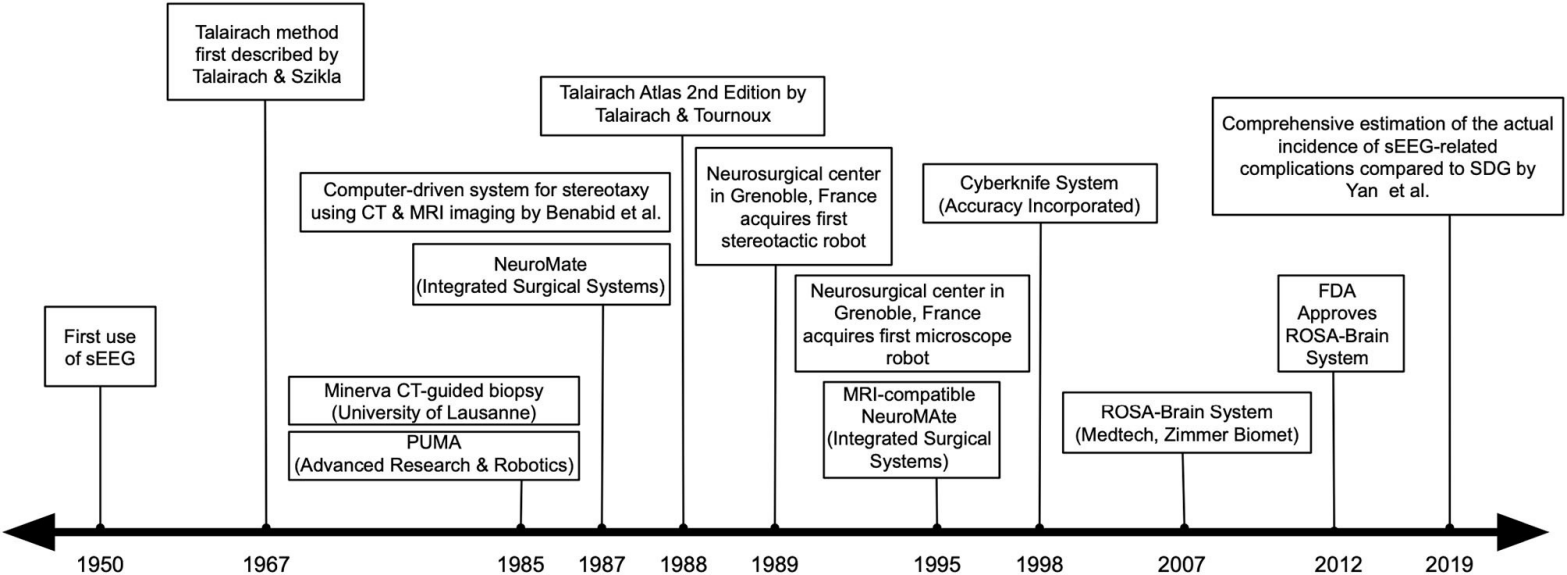
A timeline of important events in the history of robotics in sEEG surgery.

More recently, operative robotic systems for neurosurgical procedures have been increasingly adopted in the United States following the demonstration of their utility in Europe (17–22). Benabid et al. initially described a computer-driven technique for stereotaxy connected to CT and MR imaging in 1987 (5). Moreover, the neurosurgical center in Grenoble, France has utilized a stereotactic robot since 1989 and a microscope robot since 1995 for various surgical procedures (23). Their team further expanded the indications for robotic-assisted stereotaxy to include deep brain stimulation (DBS), stereoelectroencephalography (sEEG), and tumor biopsies or resections (17, 24). Finally, the ROSA-Brain system (Medtech, Zimmer Biomet) was released in 2007 and gained FDA approval in 2012 for cranial surgery (12, 25–27). The ROSA system is used typically for sEEG implantation; however, it is increasingly being applied to deep brain stimulation as well. While the ROSA system is widely utilized, there are now several other cranial robotic systems also in use for cranial surgery, including the Neuromate (Renishaw) and Renaissance (Mazor) systems (6, 16, 28–33). It is also noteworthy that much of the robust data on complications and surgical outcomes following robotic-assisted sEEG are performed using the ROSA system, which is again reflective of its widespread use specifically at large-volume epilepsy centers. Furthermore, non-robotic systems such as the FHC microtargeting epilepsy platform, may also play a role in some centers with limited patient volume or where robotic purchases may not be permissible (34, 35). These systems are less bulky and require less initial economic investment, but do not allow for a stereotactic plan which can be simultaneously modified during surgical placement. This review will describe how the advent of surgical robotics has improved the efficacy and ease of performing sEEG surgery. A timeline of important events in the history of robotics in sEEG surgery can be found in Figure 1.

## Robotic Versatility for Both Oblique and Orthogonal SEEG Trajectories

The epileptogenic zone (EZ) is commonly defined as the region of brain tissue that results in seizure freedom when removed. However, it is further described as the area of cortex which is indispensable for the generation of clinical seizures, taking into account both the anatomical location of the origin of the seizures as well as the associated regions of discharge which give rise to their accompanying clinical symptoms (36, 37). Therefore, it is essential to fully delineate the EZ prior to effective epilepsy surgery (37–39). Epileptologists attempt to identify the EZ via non-invasive methods, such as video electroencephalography (EEG) and advanced imaging studies such as magnetic resonance imaging (MRI) (40). In many instances, however, invasive monitoring is required to adequately understand and characterize the EZ (41, 42). Stereoelectroencephalography (sEEG) is a method of planning and implanting percutaneous intracerebral electrodes based upon a customized patient-specific anatamo-electro-clinical hypothesis (43). In other words, the sEEG method integrates anatomic, electrographic, and clinical information to test hypotheses regarding the localization of the EZ and has been used in Europe since the 1950s (44).

Orthogonal placement of sEEG electrodes is heavily influenced by the Talairach method (45). First described by neurosurgeons Talairach and Szikla in 1967, this method creates a standardized grid for neurosurgical procedures, whereby distances to lesions were proportional to the overall brain size (46). In 1988, a second edition of the Talairach Atlas was coauthored by Tournoux and was based upon a postmortem dissection of a human brain (47). The Talairach coordinate system is defined by making two anchors—the anterior commissure (AC) and posterior commissure (PC)—lie on a straight horizonal line in the midsagittal plane. Originally, this method used pneumoencephalograms to identify these two anatomical anchor points. Bancaud, a neurologist, collaborated with Talairach to develop a collaborative approach to sEEG implantation. Therefore, sEEG allowed not only the recording of deep brain structures but also the possibility of a three-dimensional analysis of seizure spread and distribution as well as its correlation to a patient's clinical characteristics. Orthogonal sEEG implantations are standardized to enable electrographic information to be obtained from both the cortical surface and deep targets.

One of the great advantages of robot-assisted implantation techniques is the ability to seamlessly transition between both orthogonal and oblique trajectory types using a single technique. Previously, neurosurgeons would use a Talairach frame, which provided the capability of rapid implantation of orthogonal sEEG electrodes, but was unable to place oblique electrodes (48). When the Talairach frame was used, a Leksell frame could then be used secondarily if an oblique trajectory was necessary. A recent study by Bourdillon et al., which compared the traditional orthogonal Talairach approach to a frame-based robotic technique, concluded that while both procedures are safe and sufficient, the effective accuracy (96.5 vs. 13.7%; 95% CI, −0.863 to −0.781; *p* < 0.001; *t* = −39.92) and absolute accuracy (1.15 vs. 4.00 mm; 95% CI, 2.597–3.183; *p* < 0.001; *t* = 19.73) was significantly higher in cases utilizing the robotic technique (49). These findings highlight the added value of robotics in the precision and accuracy of implantation. In contrast, the Leksell frame can be used to place both oblique and orthogonal trajectories but is limited by a need to manually configure each trajectory intraoperatively and also the Leksell frame “no-fly zone,” which sometimes necessitates replanning of trajectories intraoperatively based on Leksell frame placement (50). Robotic devices circumvent these limitations and enable a platform through which sEEG trajectories can be planned entirely before entering the operating room.

While the discussion of oblique vs. orthogonal implantation trajectories is subjective and heavily dependent on individual surgeon training and experience, it is our view that orthogonal or quasi-orthogonal implantation trajectories (in relation to the midline sagittal plane as defined by the AC–PC) are preferred for most sEEG targets. We would like to emphasize that surgical robotics allow for the safe and accurate implantation of sEEG electrodes in any direction; however, oblique trajectories play a crucial role for some targets (see below). There are three primary conceptual justifications, in our view, for favoring orthogonal implantations for the majority of sEEG trajectories:

1. Coverage volume reasons: Numerous functional networks are distributed and organized along an orthogonal orientation vs. the mid-sagittal plane. For example, the orientation of the cortical and subcortical perisylvian areas are principally distributed along a rostral–caudal orientation. Therefore, systematic orthogonal electrode implantation facilitates an understanding of the target structure(s) and also its interaction with surrounding brain pathways. While there are many networks in the brain that are not organized orthogonally, many relevant to the mesial temporal lobe, for example, can be investigated in an orthogonal approach to record activity of the amygdala or hippocampus, while more superficial trajectories can record from the temporal neocortex. In relation to SEEG electrode implantation, the Talairach method also aims to successively place anteroposterior and dorsoventral depth electrodes, thus obtaining broad electrophysiological coverage that reconstitutes the three-dimensional brain volume. In addition, this technique remains uniquely suited for the rational investigation of longitudinal and transversal cortical connections. If oblique trajectories are applied as the primary manner of exploration, this three-dimensional understanding may be overlooked or underrepresented.
2. Surgical reasons: The three-dimensional anatomical definition of the EZ is conceptualized in orthogonal planes (axial, coronal, and sagittal) during surgical resections (51). Thus, neurosurgeons typically appreciate neuroanatomy in orthogonal orientations that are constructed within the surgeon's mind, as these orthogonal planes and their anatomic relationships are constant and predictable and routinely parallel with imaging studies. Upon introduction of an oblique plane, this necessary predictability is lost, and the anatomical relationships between structures is increasingly obscured (52). With the advent of surgical robotics, oblique trajectories have become increasingly prevalent. This ultimately results in significantly increased complexity in interpreting SEEG recordings. Finally, a comprehensive neuroimaging pipeline allows for the direct visualization of sEEG anatomical targets and electrodes. The quality of imaging studies and the overlay between a fiducial-based scan and the planning scan allows the stereotactic robot arm to maximize precision via an accurate registration, safely place sEEG electrodes, and provide for meaningful interpretation of clinical information. As the technology evolves, image registration may eventually become an automated process; however, it will still rely on selection of fiducials until real-time imaging feedback can be incorporated into the surgical workflow.
3. Technical reasons: Orthogonal implantations may be safer and more precise when compared to oblique trajectories (53). Oblique trajectories in relation to the skull may generate lead deflections, which may factor into placement inaccuracies (such as epidural electrodes, for example) or predispose a patient to complications, such as intracranial hemorrhage. In addition, long oblique trajectories (e.g., the medial–lateral insular trajectory) may have decreased accuracy due to long span. Orthogonal implantations are shorter and moreover less prone to deflections.

Oblique implantations are conducted in specific situations, where the targeted cortical areas are truly and more efficiently explored via such trajectories, as described:

1. Ventromedial prefrontal cortex (vmPFC): Significant portions of vmPFC, which includes the gyrus rectus and the orbital gyri, can be difficult to investigate via standard orthogonal sEEG trajectories. The bony structure of the orbital cavity necessitates oblique trajectories. As mentioned, attempts at low orthogonal approaches to the gyrus rectus may also lead to a subdural deflection of the sEEG electrode. The preferred implantation method for this region is obtained via oblique orientation electrodes with an entry point located in the frontal areas (at the hair line) in converging orientation and targeting the most posterior and medial aspect of the vmPFC in the gyrus rectus.
2. Dorsal frontal and parietal areas: The dorsal frontal areas are also challenging to investigate via orthogonal SEEG trajectories. The skull is curved in this topography, thus making orthogonal implantations imprecise and perhaps less safe (53). Oblique implantations are more suitable when placed along the coronal plane while preserving orthogonality in the sagittal plane. The dorsal parietal areas are equally as difficult for orthogonal electrode implantation for similar reasons. In these situations, oblique SEEG trajectories that are nearly perpendicular to the skull are preferred to avoid deflection.
3. Skull defects preventing orthogonal trajectories: Skull defects, such as from prior surgical intervention, may prevent typical lead placement if the bony defect is in the orthogonal projection of the intended target. In this scenario, oblique implantations are preferred and perhaps the only alternative. This may further apply to trajectories where bone may be too thin to accommodate an anchor post, or in the presence of air cells, bony emissary vessels, etc. However, there are unique situations in which oblique trajectories are warranted. For example, in some cases of temporal corticectomy, an oblique trajectory may be required to reach mesial structures.
4. Insula: The insular cortex can be explored with either orthogonal or oblique SEEG trajectories (54, 55). However, a medial to lateral oblique trajectory maximizes gray matter coverage of the insular gyri. Trajectories originating from the frontal bone can target the anterior short gyri and conversing a parietal bone entry site can be used to approach the posterior long insular gyri. A combination of both trajectories is commonly used, depending on the pre-implantation hypothesis. These oblique trajectories maximize insular coverage by providing several contacts within the insula, whereas orthogonal approaches provide only one or two insular electrodes per trajectory (56). The disadvantage of oblique approaches is the lack of opercular coverage. With an orthogonal approach, a single SEEG electrode may explore the insula with its distal contacts and also explore the adjacent opercular cortex with its most superficial contacts. Since the insulo-opercular regions are often involved together in the EZ, the simultaneous evaluation of both cortical areas has distinct advantages in understanding the organization of the EZ and provides a functional assessment of the opercular areas, which may ultimately require resection to gain surgical access to the insular cortex. However, this subtle disadvantage of oblique investigations can be overcome with the addition of orthogonally placed electrodes in the opercular areas in addition to oblique insular electrodes, albeit this approach requires added electrodes.

## Complications and Patterns of Use in SEEG Implantation: Contributions of Robotics

While different invasive monitoring techniques offer distinct philosophical approaches, as well as unique advantages and disadvantages, sEEG stands apart as a less invasive approach (57). Furthermore, sEEG provides an exclusive opportunity to sample and record from deep cortical structures with unparalleled accuracy to provide surgeons and clinicians with high-powered, three-dimensional mappings of epileptic networks that are used to meticulously guide resection (58). As the use of sEEG for invasive monitoring becomes increasingly popular, both within the United States and across the globe, its relative safety and efficacy has been well-documented and its rate of complications are reported to be the lowest amongst all methods of invasive monitoring in both adult and pediatric patient populations (59–64). Though sEEG has been in use in France since the 1950s, the advent of robotics and recent neuroimaging techniques have led to its proliferation and acceptance throughout North America (65).

The largest sEEG series ever reported, by Cardinale et al., described the 20-year single-institution experience of seizure freedom rates, outcome predictors, and complication rates from 742 sEEG procedures in 713 patients conducted between May 1996 and July 2018 (66). Seizure freedom outcomes were compared to 1,128 patients who underwent surgical intervention after non-invasive evaluation. Furthermore, 185 of the sEEG cases (25.9%) were pediatric patients with the average total cohort age of 26.2 ± 11.8 years. The institutional surgical workflow consisted of the traditional Talairach approach until 2009, after which time the center adopted an image-guided workflow utilizing 3D imaging and robotic assistance (3DIRA). Among sEEG patients, resective surgery was indicated in 79.9% of the total cohort, with 59.4% of patients ultimately achieving seizure freedom at 2 years. With regards to medical and procedural complications, these were present in 13 (1.8%) procedures of which 4 (0.5%) were classified as major events (i.e., one death, two permanent contralateral hemiplegic conditions, and one unilateral leg compartment syndrome with permanent deficit). Although statistically insignificant, it is noteworthy that the overall complication rate was lower following implementation of the 3DIRA workflow (0.9 vs. 2.4%; *p* = 0.16), and no major procedure-related complications were reported out of 5,181 3DIRA-implanted electrodes. These findings substantially support the efficacy and safety of sEEG in both adult and pediatric patients. Furthermore, the decreased rate of complications following implementation of a 3DIRA workflow emphasizes the value and utility of new robotic technologies in neurosurgery.

In 2016, Mullin et al. published a meta-analysis of observational data describing the rates of sEEG complications in 2,624 patients aged between 1 and 69 years (average age, 24 years) from 30 previously published studies (62). Additionally, the review included 124 pediatric patients (4.7%) from four previously published studies examining the efficacy and safety of sEEG in children (67–70). The pooled prevalence rate of 1.3% (95% CI 0.9–1.7%) for all complications demonstrates a remarkably low complication rate in sEEG surgery. While the most common risk is hemorrhagic complications (pooled prevalence 1.0%, 95% CI 0.6–1.4%), the hemorrhage rate was significantly lower when compared to a meta-analysis of patients monitored with subdural grid electrodes (SDG; pooled prevalence 4.0%, 95% CI 3.2–4.8%) (61). Furthermore, a lower overall rate of infection in sEEG monitored patients (pooled prevalence 0.8%, 95% CI 0.3–1.2%) was demonstrated when compared to the reported infection rate (pooled prevalence 2.3%, 95% CI 1.5–3.1%) in the previously mentioned meta-analysis. The overall pooled prevalence of either transient or permanent neurological deficits was 0.6% (95% CI, 0.2–1.0%); however, it was noted that the causes of permanent neurological deficit were not always attributable to sEEG. Of the 2,624 patients in the pooled meta-analysis, there were five reported mortalities: two from intracerebral hemorrhage (ICH), two from preimplantation ventriculography (which is no longer performed routinely at most centers), and one from severe cerebral edema from a likely underlying metabolic derangement. The meta-analysis also noted a total of 11 hardware complications, including one patient who required an additional craniotomy for removal of a retained electrode.

When comparing the complication rates of the two most common methods of invasive monitoring, sEEG and SDG, the relative safety and efficacy of sEEG becomes increasingly clear. A systematic review published by Yan et al. in 2019 examined rates of epilepsy surgery-associated morbidity and subsequent seizure freedom in patients with drug-resistant epilepsy (DRE) monitored with either sEEG or SDG (59). The review included 48 observational studies that captured 1,973 sEEG patients and 2,036 SDE patients, of which 29 examined both adult and pediatric patients and 8 were pediatric-only studies. While none of the included studies performed direct head-to-head comparisons between the two monitoring techniques, sEEG was associated with 4.8% morbidity compared to a rate of 15.5% with SDG (WMD, −10.6%; 95% CI, −11.6–19.6%; *p* = 0.001). Reported complications included hemorrhage, infection, cerebrospinal fluid (CSF) leak, lead fracture, transient and permanent neurological deficits, and medical complications. Rates of subdural and epidural hematoma (0.7% sEEG; 3.4% SDG; WMD, −2.6%; 95% CI, −2.8 to −2.4%; *p* = 0.01), cerebrospinal fluid leak (0% sEEG; 0.6% SDG; WMD, −1.0%; 95% CI, −1.1 to −0.9%; *p* = 0.01), lead fracture (0.4% sEEG; 1.0% SDG; WMD, −0.5%; 95% CI, −0.7 to −0.2%; *p* = 0.01), transient neurological deficit (1.9% sEEG; 5.7% SDG; WMD, −1.4%; 95% CI, −1.7 to −1.1%; *p* = 0.01), and medical complications (0.7% sEEG; 2.6% SDG; WMD, −1.4%; 95% CI, −1.7 to −1.2%; *p* = 0.01) were significantly lower among sEEG patients compared to SDG. The rate of infection was also significantly lower among sEEG patients (0.9% sEEG; 1.6% SDG; WMD, −1.6%; 95% CI, −1.7 to −1.5%; *p* = 0.01). Although sEEG can be technically difficult in very young children (i.e., before the age of 2 years), it provides a means of extended recording time coupled with a lower risk of infection compared to SDG (71). While intraparenchymal hemorrhage was significantly more common in sEEG (2.3% sEEG; 1.4% SDG; WMD, 1.5%; 95% CI, 1.4 to −1.7%; *p* = 0.01), the results suggest an overall lower complication profile compared to SDG. The pooled prevalence of mortality was 0.2% among sEEG patients and 0.4% among SDG (WMD, −10.6%; 95% CI, −11.6 to −9.6%; *p* = 0.01) with all mortalities attributed to the method of invasive monitoring itself.

Additional benefits of sEEG with regards to low complication rates can be seen at the individual patient level. A recently published individual patient data (IPD) meta-analysis by Remick et al. was the first to simultaneously examine individual patient phenotypes and their outcomes following invasive monitoring with either sEEG or SDG (63). The review analyzed 595 patients from 33 studies, of which 15 examined both adult and pediatric patients and nine included pediatric patients only. Morbidities such as infection, hemorrhage, and transient and permanent neurological deficits were used as dependent variables in a regression analysis aimed at identifying their associations with patient phenotypes. The results indicate that clinical profiles of patients undergoing sEEG significantly differ from their SDG counterparts. For example, sEEG was a dominant contributor to patient phenotypes associated with low morbidity, while patient phenotypes involving multiple subpial transections, anterior temporal lobectomy, amygdalectomy, and hippocampectomy disproportionately contributed to greater morbidity and were strongly colinear with SDG. As a result, complication rates may be associated with the unique epileptic etiologies that invasive monitoring is used to explore. The authors conclude that while sEEG is associated with a lower rate of resection (82.0%; 95% CI, 78.8–84.2%) compared with SDG (92.7%; 95% CI, 91.1–94.4%; *p* = 0.0002), the clinical phenotypes of sEEG patients were also associated with lower rates of complication, suggesting that the nature of the invasive monitoring technique itself may contribute to patient morbidities. As a minimally invasive approach, sEEG may provide patients with a lower risk approach to successful localization of the EZ.

Finally, there may be spatial and temporal trends in sEEG utilization that contribute to complications in its use. For example, sEEG electrodes were first used to study epilepsy in France during the 1950s; however, the technique did not emerge in practice in the United States until the mid-1970s, where it has been slow to gain popularity (72). While sEEG usage has exponentially increased in recent decades, it is still considered a relatively novel technique when compared to traditional North American methods such as SDG (64). As a result, differences in both institution- and surgeon-level education, training, and experience may contribute to observed rates of complications and decrease over time as sEEG becomes a more widely utilized approach. Furthermore, while modern robotic placement is gaining traction as a valuable enhancement to the precision and accuracy of traditional stereotaxy, many centers continue to utilize manual frame-based and frameless techniques for electrode insertion.

While many of the studies discussed in this review are reflective of adult data, a great number included children in their analyses. This is not surprising as pediatric cases represent a large proportion of sEEG implantations, especially at high volume epilepsy centers. However, the safety profile of robotic sEEG usage in children is comparable to adults in pediatric-only studies (48, 70, 71, 73–76). Furthermore, some studies have examined differences in the utility of frameless robotic technique as opposed to the traditional Talairach frame approach, suggesting that pediatric patients specifically benefit from the swift precision and accuracy that is gained through the use of surgical robots in sEEG surgery. A recent observational study described the technical aspects of and comparison between frameless robot-assisted vs. Talairach frame-based sEEG in 17 children with DRE at an institution with over 30 years of sEEG experience (48). The authors report that, while there were no significant differences in complication rates regardless of a robotic approach, the frameless robot-assisted technique was more efficient, as it required less operating room time and time under anesthesia.

## Conclusion

The contributions of robotics to the safety and efficacy of invasive monitoring in epilepsy surgery have grown substantially in recent decades. Although sEEG has been in use in France since the 1950s, the advent of robotics and recent neuroimaging techniques have led to its proliferation and acceptance throughout North America. The traditional Talairach frame approach provided the capability of rapid implantation of orthogonal sEEG electrodes; however, it falls short of allowing placement of oblique electrodes. One of the great advantages of robot-assisted implantation techniques is the ability to seamlessly transition between both orthogonal and oblique trajectory types using a single technique. Furthermore, while there are a variety of factors that contribute to both the rates and types of complications observed in sEEG patients, sEEG surgery demonstrates an inherently low complication profile, especially when compared to traditionally held methods of invasive monitoring, such as SDG.

## Author Contributions

AF, MR, and TA equally contributed to the literature review, drafting, and final version of the manuscript in its entirety. All authors read and approved the final manuscript.

## Conflict of Interest

The authors declare that the research was conducted in the absence of any commercial or financial relationships that could be construed as a potential conflict of interest.
